# Tearable and Fillable Composite Sponges Capable of Heat Generation and Drug Release in Response to Alternating Magnetic Field

**DOI:** 10.3390/ma13163637

**Published:** 2020-08-17

**Authors:** Koichiro Hayashi, Atsuto Tokuda, Jin Nakamura, Ayae Sugawara-Narutaki, Chikara Ohtsuki

**Affiliations:** 1 Department of Biomaterials, Faculty of Dental Science, Kyushu University3-1-1, Maidashi, Higashi-ku, Fukuoka 812-8582, Japan; 2 Department of Materials Chemistry, Graduate School of Engineering, Nagoya University, Furo-cho, Chikusa-ku, Nagoya 464-8603, Japan; tokuda.atsuto@f.mbox.nagoya-u.ac.jp (A.T.); nakamura@chembio.nagoya-u.ac.jp (J.N.); ayae@energy.nagoya-u.ac.jp (A.S.-N.); ohtsuki@chembio.nagoya-u.ac.jp (C.O.)

**Keywords:** magnetic nanoparticles, composite, DDS, hyperthermia, collagen

## Abstract

Tearable and fillable implants are used to facilitate surgery. The use of implants that can generate heat and release a drug in response to an exogenous trigger, such as an alternating magnetic field (AMF), can facilitate on-demand combined thermal treatment and chemotherapy via remote operation. In this study, we fabricated tearable sponges composed of collagen, magnetite nanoparticles, and anticancer drugs. Crosslinking of the sponges by heating for 6 h completely suppressed undesirable drug release in saline at 37 °C but allowed drug release at 45 °C. The sponges generated heat immediately after AMF application and raised the cell culture medium temperature from 37 to 45 °C within 15 min. Heat generation was controlled by switching the AMF on and off. Furthermore, in response to heat generation, drug release from the sponges could be induced and moderated. Thus, remote-controlled heat generation and drug release were achieved by switching the AMF on and off. The sponges destroyed tumor cells when AMF was applied for 15 min but not when AMF was absent. The tearing and filling properties of the sponges may be useful for the surgical repair of bone and tissue defects. Moreover, these sponges, along with AMF application, can facilitate combined thermal therapy and chemotherapy.

## 1. Introduction

Surgery is a standard treatment for removing bone and soft tissue tumors [1,2]. To reconstruct the defects formed by surgery, restorative materials are frequently implanted into the defect site [3,4,5]. The use of restorative materials that can release anticancer drugs may play a role in the prevention of the recurrence of tumors as well as the reconstruction of the defects [6,7,8,9].

To date, porous materials have been developed as implantable carriers for drug delivery systems (DDS) [10,11,12]. Notably, sponge-like materials are promising implantable carrier materials for DDS because they easily fill defects owing to their tearable and flexible properties [13,14,15,16,17]. To date, various sponge-like materials composed of chitosan, silk, and gelatin have been fabricated by freeze-drying and electrospinning methods [13,14,15,16,17]. However, in many cases, drug release from implantable carriers is difficult to control because they are spontaneously dissolved in the body [17]. Therefore, researchers have tried to actively control drug release by developing implantable carriers with responsiveness to stimuli such as light [18] and electric fields [19,20]. Although these stimuli have the advantage of being switchable, they cannot penetrate deep into the body. Therefore, the use of these stimuli is considered challenging for the active control of drug release.

Although chemotherapy controlled by DDS is a promising tumor treatment, the effectiveness of anticancer drugs depends on the cancer type and stage [20]. In contrast, treatments such as thermal therapy show therapeutic effects regardless of cancer type and stage [21,22,23]. It has been reported that the combination of chemotherapy and thermal treatment, enhances treatment efficacy [24,25]. Notably, magnetic nanoparticles and anticancer drug-loaded materials can be used in a way to exploit the heat generated by magnetic nanoparticles in response to alternating magnetic field (AMF) exposure [26,27,28,29], which can subsequently act as a trigger for releasing drugs from the materials, achieving a remotely controllable on-demand administration of combined chemotherapy and thermal treatment [30,31,32,33,34].

In this study, we synthesized magnetic nanoparticles and anticancer drug-loaded collagen sponges and evaluated their ability to generate heat and drug release behavior. Furthermore, the therapeutic efficacy of combined chemotherapy and thermal treatment through the use of the composite sponge and AMF application was evaluated using in vitro assays.

## 2. Experimental Section

### 2.1. Synthesis of Magnetic Nanoparticles (MNPs)

The MNPs were prepared using a previously reported method [35,36]. Briefly, iron (III) acetylacetonate, Fe(acac)_3_, (Nihon Kagaku Sangyo, Tokyo, Japan) was dissolved in ethanol. Subsequently, hydrazine monohydrate (Kishida Chemical, Osaka, Japan) and distilled water were added to the Fe(acac)_3_ solution, and then the mixture was stirred at 78 °C for 24 h. The magnetic properties of iron oxide (magnetite and/or maghemite) nanoparticles were controlled by adjusting the Fe(acac)_3_ concentration and the amounts of hydrazine monohydrate and distilled water added to the mixture. The Fe(acac)_3_ concentration and the amounts of hydrazine monohydrate and distilled water used are shown in Table S1. The MNPs were collected by centrifugation of the solution at 10,000 rpm for 10 min. The obtained MNPs were washed with ethanol and distilled water three times, respectively.

### 2.2. Fabrication of the MNPs and Anticancer Drug-Loaded Collagen Sponge

The anticancer drug we used was doxorubicin hydrochloride (DOX; Tokyo Chemical Industry, Tokyo, Japan). For the fabrication of the MNPs and DOX-loaded collagen sponge (MDC sponge), MNPs with the highest saturation magnetization (MNPs-8) were used. The MNPs (3.5 mg) and DOX (85 μg) were mixed with a type-I collagen solution (5 mg/mL, Nitta Gelatin, Osaka, Japan), and the MDC sponge was fabricated by freeze-drying the mixture at −80 °C for 48 h. To control DOX release from the MDC sponge, the MDC sponge was crosslinked by heat treatment at 140 °C for 1.5, 6, or 24 h under vacuum. To confirm that the MDC sponge contained DOX using Fourier-transform infrared (FTIR) spectroscopy, a collagen sponge loaded with MNPs (without DOX; MC sponge) was fabricated.

### 2.3. Characterization of the MNPs and MDC sponge

The microstructures of the MNPs and MDC sponge were observed using a transmission electron microscope (TEM; JEM-2100Plus, JEOL, Tokyo, Japan) and a scanning electron microscope (SEM; JSM-5600, JEOL, Tokyo, Japan). The crystal phases of the MNPs and MDC sponge were confirmed using X-ray diffraction (XRD; RINT-2100/PC, Rigaku, Tokyo, Japan). The crystallite size of the MNPs was calculated using Scherrer’s equation and the 311-diffraction peak. The FTIR spectra were obtained using an FTIR spectrometer (FT/IR-6100, JASCO, Tokyo, Japan). The inorganic and organic percentages of the MDC sponge were measured using thermogravimetric and differential thermal analysis (TG-DTA; DTG-60AH, Shimadzu, Kyoto, Japan). The magnetic properties of the MNPs and MDC sponge were measured at room temperature using a vibrating sample magnetometer (VSM; BHV55, Riken Denshi, Tokyo, Japan).

### 2.4. Heat Generation Properties of the MNPs

The MNPs with the highest saturation magnetization (MNPs-8) were uniformly suspended in distilled water by sonication (50 W, 20 kHz, 30 s) using an ultrasonic oscillator (VCX-50PB, Ieda Trading, Tokyo, Japan) at a concentration of 1 mg/mL. The MNPs were suspended for a few hours at least and no precipitation was observed. The suspension was placed inside the coil of an induction heater (Easy Heat, Alonics, Tokyo, Japan). Subsequently, the suspension was exposed to a magnetic field of 74 Oe and a frequency of 216 kHz for 10 min. The temperature of the suspension was measured every 30 s using an infrared thermal imaging camera (InfReC G100EX, Nippon Avionics, Tokyo, Japan). The heat generation properties of MNPs were assessed based on the specific absorption rate (SAR) of the particles. SAR was calculated using the following equation:

(1) $$\mathrm{SAR}=\frac{mC}{m_{Fe_{3}O_{4}}}\times \frac{dT}{dt},$$

where, C is the specific heat of water (4.2 J/(g·K)); *m* is the mass of the sample; $m_{Fe_{3}O_{4}}$ is the mass of the MNPs in the sample; *T* is the temperature; *t* is the application time of the AMF; and d*T*/d*t* is the slope of the curve of temperature vs. application time of the AMF in the first 30 s [37].

### 2.5. DOX Release from the MDC Sponge Without the AMF Application

An untreated MDC sponge (4.4 mg) and an MDC sponge (4.4 mg) that was crosslinked for 1.5, 6, or 24 h, were immersed in phosphate-buffered saline (PBS, 1.0 mL) at 37 °C and 45 °C. The UV-vis spectra of the supernatant (400‒800 nm of wavelength range) were measured using UV-vis spectroscopy (V-670 spectrophotometer, JASCO, Tokyo, Japan). The amount of released DOX was estimated using the Beer–Lambert law based on the absorbance at 480 nm.

### 2.6. Heat Generation Properties of MDC Sponge

An MDC sponge crosslinked for 6 h (11 mg) was immersed in a cell culture medium (1.3 mL) and exposed to AMF (magnetic field of 74 Oe and frequency of 216 kHz) for 20 min using an induction heater. The temperature of the MDC sponge in the cell culture medium was measured every 30 s using an infrared thermal imaging camera.

### 2.7. Control of Heat Generation and DOX Release by Switching the AMF on and off

An MDC sponge crosslinked for 6 h was immersed in a cell culture medium, and the system temperature was kept at 37 °C. The MDC sponge was exposed to AMF (magnetic field of 74 Oe of magnetic field and frequency of 216 kHz) at 15-min intervals by switching the AMF on and off. The solution temperature during AMF application was measured using an infrared thermal camera. The amount of DOX released from the MDC sponge every 15 min was estimated using the Beer–Lambert law based on the absorbance at 480 nm, which was measured using UV-vis spectroscopy. 

### 2.8. Destructive Ability of the MDC Sponge on HeLa Cells in the Presence of an AMF

HeLa cells (Riken, Tsukuba, Japan) were cultured in Dulbecco's modified Eagle's medium (DMEM; Fujifilm Wako Pure Chemical, Osaka, Japan) supplemented with fetal bovine serum (FBS; final concentration 10%, Sigma Aldrich, MO), MEM non-essential amino acids solution (final concentration 1%, Fujifilm Wako Pure Chemical), and a penicillin–streptomycin solution (final concentration 1%, Fujifilm Wako Pure Chemical). Cells were seeded at a density of 2.5×10^4^ cells per well in a 24-well plate and cultured under 5% CO_2_ at 37 °C for 24 h. Cells were enumerated using a cell counter (Cell Counting Kit-8, Dojindo Laboratories, Kumamoto, Japan). The MDC sponge crosslinked for 6 h (3.5 mg) was placed in a well and exposed to AMF (magnetic field of 74 Oe and frequency of 216 kHz) for 15 min using the induction heater. Cell viability was measured using cytotoxicity assays and a tetrazolium salt (CCK-8 assay system, Takara Bio, Shiga, Japan) at days 3 and 5 after AMF application. In the CCK-8 assay, the absorbance at 460 nm was measured using a microplate reader (Epoch 2, BioTek Instrument, VT). As a control, we measured the cell viability of the non-treated cells and cells cultured with the MDC sponge in the absence of the AMF application. Significant differences were estimated by multiple comparisons between groups using a general multiple comparison method, the Tukey–Kramer method. *p* < 0.05 was considered statistically significant.

## 3. Results and Discussion

### 3.1. The Structure, Magnetic Properties, and Heat Generation Ability of the MNPs

The XRD patterns showed that all the MNPs were composed of magnetite and/or maghemite (Figure S1). The crystallite size of the MNPs was increased with increasing Fe(acac)_3_ concentration and the additive amounts of hydrazine monohydrate and distilled water (Figure S2). We have previously demonstrated that the crystallite size increased by increasing the amount of the iron source. Furthermore, hydrolysis of the iron complex was promoted by increasing the amounts of hydrazine and water, resulting in an increase in crystallite size. Thus, the results of this study are consistent with those of our previous reports [31].

The magnetization curves of all the MNPs showed neither coercivity nor remnant magnetization (Figure S3), indicating that the MNPs were superparamagnetic. It has been reported that MNPs less than 10 nm in diameter exhibit superparamagnetic properties [38]. As the crystallite sizes of all the MNPs in our study were less than 10 nm in diameter, all the MNPs exhibited superparamagnetic properties. Furthermore, the magnetization of the MNPs increased when the Fe(acac)_3_ concentration (Figure S3A) and the additive amounts of hydrazine monohydrate (Figure S3B) and distilled water (Figure S3C) increased. Thus, the magnetization of the MNPs increased as the crystallite size increased. As the MNPs-8 had the highest magnetization (76.8 emu/g) at 15 kOe, they were used to fabricate the MDC sponges.

The MNPs-8 were uniformly suspended in distilled water and generated heat in response to AMF exposure (74 Oe and 216 kHz), raising the water temperature from 28.5 to 56.8 °C for 10 min (Figure S4). The SAR was 70.6 W/g. Hergt et al. reported that the SAR of Endorem, a magnetic resonance imaging contrast agent consisting of 6-nm-MNPs, was < 0.1 W/g at 300 kHz and 82 Oe [39]. Timko et al. reported that the SAR of MNPs with a diameter of 10–140 nm enveloped by a biological membrane consisting of phospholipids and specific proteins was 171 W/g at 750 kHz and 63 Oe [40]. Drake et al. reported that the SAR of Gd-doped iron oxide was 36 W/g at 52 kHz and 246 Oe [41]. Generally, SAR is proportional to the frequency and the square of the amplitude of the magnetic field [42]. Thus, the MNPs-8 had higher heat generation abilities than the reported materials. We have previously demonstrated that the dead layer of MNPs synthesized using the same method used in this study was thin, providing high heat generation abilities [43].

### 3.2. The Structure of the MDC Sponges

**Figure 1:**
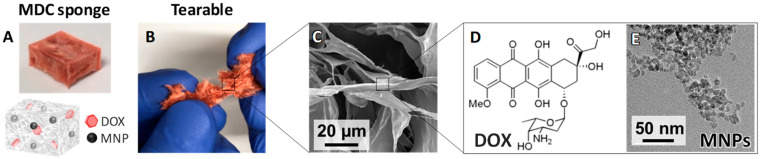
Characteristics of the MNPs and DOX-loaded collagen (MDC) sponges. (**A**) Photograph of the MDC sponge and illustration showing its structure. (**B**) Photograph showing that the MDC sponge is tearable. (**C**) SEM images of the MDC sponge. (**D**) Chemical structure of DOX. (**E**) TEM images of MNPs-8.

The MDC sponges were flexible and tearable (Figure 1A,B), characteristics that facilitate the filling of the defects formed by surgery. The MDC sponges were primarily composed of collagen fibers (Figure 1C), which contained DOX and MNPs (Figure 1D,E). The crosslinking of collagen reportedly impacts mechanical properties, such as the elastic modulus and elongation [44,45]. Owing to this crosslinking effect, the handleability for filling the MDC sponges into the defects seems to be improved.

**Figure 2:**
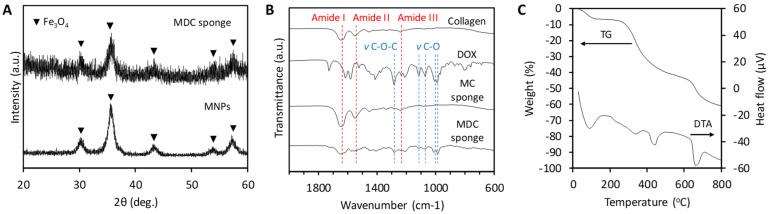
The diffraction peaks of magnetite were detected in the X-ray diffraction (XRD) patterns (**A**) XRD patterns of the magnetite nanoparticles (MNPs) and DOX-loaded collagen (MDC) sponge and MNPs. (**B**) FTIR spectra of collagen, DOX, MC sponge, and MDC sponge. (**C**) Thermogravimetric and differential thermal analysis (TG-DTA) curves of MDC sponge.

The diffraction peaks of magnetite and/or maghemite were detected in the XRD patterns (Figure 2A). In the FTIR spectra of the MC and MDC sponges (Figure 2B), absorption bands attributable to amide groups in collagen were detected at 1650 cm^−1^ (amide I band), 1560 cm^-1^ (amide II band), and 1235 cm^−1^ (amide III band) [46]. Furthermore, in the spectra of the MDC sponges, bands attributable to DOX were detected at 1283 cm^−1^ (*ν* C-O-C), 1114 cm^−1^ (primary alcohol, *ν* C-O), 1070 cm^−1^ (secondary alcohol, *ν* C-O), and 988 cm^−1^ (tertiary alcohol, *ν* C-O) [47]. The XRD and FTIR results demonstrated that the MDC sponges contained MNPs and DOX in the collagen matrix. The TG-DTA curves showed weight losses due to dehydration in the range of 30–100 °C and due to the burnout of organics in the range of 200–800 °C (Figure 2C). The TG result revealed that the magnetite percentage was 38.7 wt %.

### 3.3. Control of DOX Release from the MDC Sponges in the Absence of AMF Application

To control the DOX release from the MDC sponge in the absence of AMF exposure, the MDC sponge was crosslinked by heat treatment at 140 °C for 1.5, 6, or 24 h under vacuum. The crosslink density was calculated by the area ratio between the P1 band due to the free amino group (1541 cm^−1^) and the invariant P2 band, ascribed to –CH_2_ in-plane bending vibration (1456 cm^-1^) in the FTIR spectrum [48]. The P2/P1 values were calculated (Figure S5B) based on the FTIR results of the MDC sponges before and after crosslinking for 1.5, 6, and 24 h (Figure S5A). The P2/P1 values increased as the crosslinking time increased (Figure S5B). Thus, the crosslink density increased as a result of the increase in crosslinking time.

**Figure 3:**
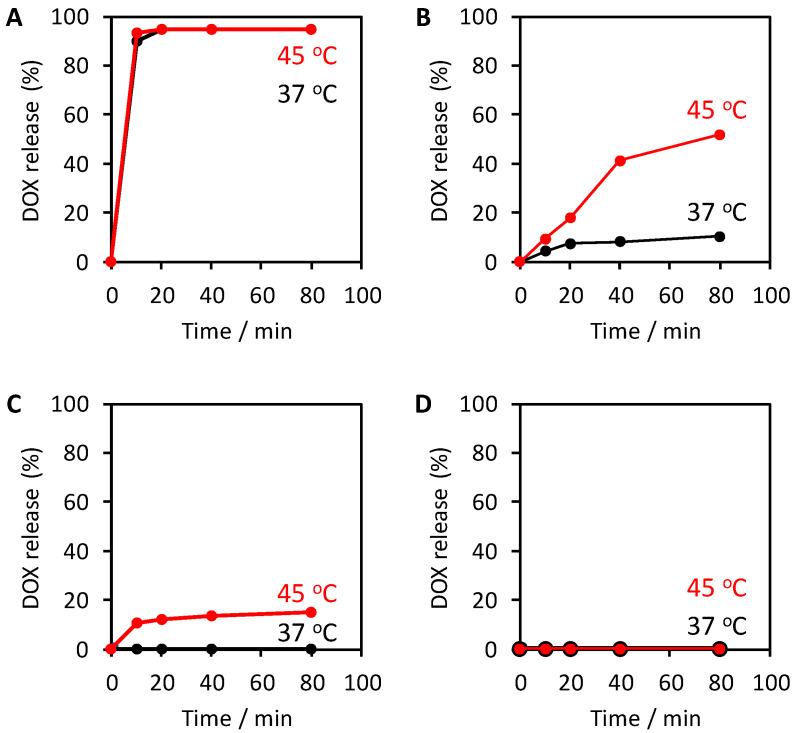
DOX release from the MDC sponge without crosslinking treatment (**A**) and with 1.5 h (**B**), 6 h (**C**), and 24 h (**D**) crosslinking treatment in phosphate-buffered saline (PBS) and the absence of alternating magnetic field (AMF) at 37 °C and 45 °C.

In the MDC sponges that were not crosslinked (Figure 3A), almost all the DOX was released from the sponges within 10 min after immersion in PBS both at 37 °C (body temperature) and 45 °C (thermal treatment temperature). In contrast, in the sponges that were crosslinked for 1.5 h, rapid DOX release immediately after immersion was suppressed (Figure 3B). However, a gradual DOX release still occurred at 37 °C, while heating at 45 °C prompted DOX release. Furthermore, a 6 h crosslinking treatment of the MDC sponges led to the complete suppression of DOX release at 37 °C and a gradual DOX release at 45 °C (Figure 3C). DOX was not released from the MDC sponges that were crosslinked for 24 h either at 37 °C or 45 °C (Figure 3D). The crosslinking of collagen reportedly reduces the swelling [44,45]. Thus, this crosslinking effect may allow for the prevention of DOX release from the MDC sponges that underwent crosslinking treatment at 37 °C. The above results demonstrated that an on-demand release of DOX using an exogenous trigger such as AMF exposure could be achieved by crosslinking the MDC sponges for 6 h.

### 3.4. The Magnetic Properties and Heat Generation Ability of the MDC Sponges

**Figure 4:**
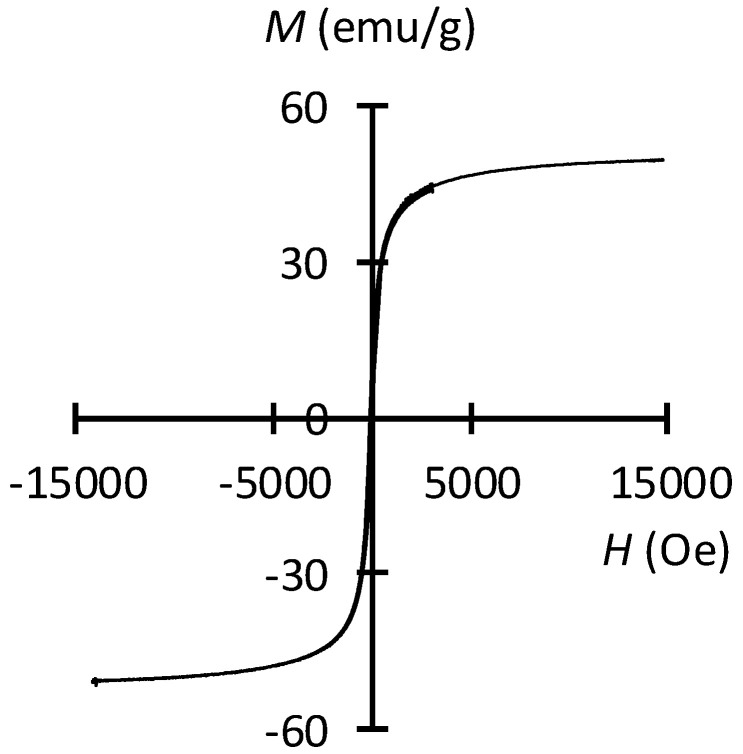
Magnetization curve of the MDC sponge at room temperature.

Consistent with the MNPs findings, the MDC sponges had neither coercivity nor remnant magnetization at room temperature and exhibited superparamagnetic properties (Figure 4). The saturation magnetization of the MDC sponges was 44.8 emu/g, which was very close to the value (44.4 emu/g) that is required to obtain thermal treatment efficacy [49].

**Figure 5:**
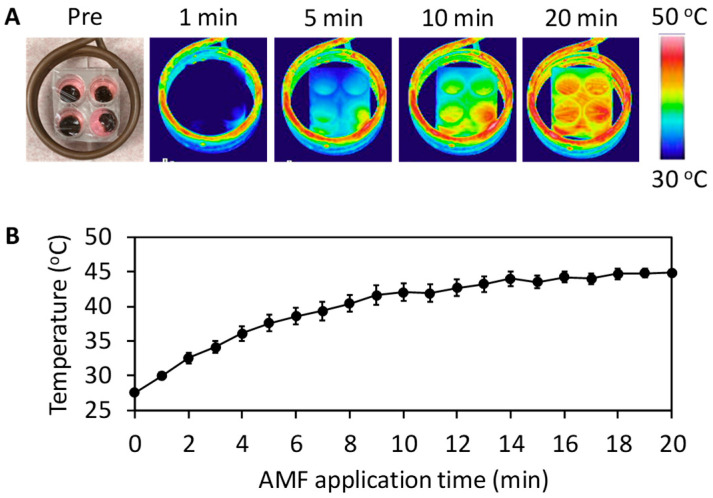
(**A**) Photograph and thermal images of MDC sponges in the cell culture medium before and during AMF application. (**B**) Change in medium temperature with AMF application time.

The MDC sponges generated heat in the cell culture medium in response to AMF exposure (Figure 5A) and heated the cell culture medium from 27.5 to 44.9 °C for 20 min (Figure 5B).

### 3.5. Control of DOX Release from the MDC Sponges by Switching the AMF on and off

**Figure 6:**
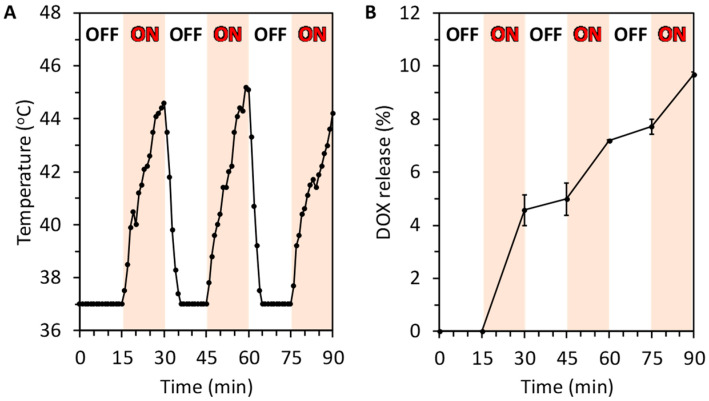
(**A**) Temperature change in MDC sponge-immersed in the cell culture medium in response to switching the AMF on and off. (**B**) DOX release from the MDC sponge when AMF was repeatedly applied and removed.

The MDC sponges were exposed to AMF at 15-min intervals by switching the AMF on and off in the cell culture medium. The MDC sponges responded immediately to AMF exposure and generated heat, increasing the medium temperature to 45 °C within 15 min (Figure 6A). The MDC sponges stopped generating heat immediately after the AMF was switched off, resulting in a rapid fall in the medium temperature to 37 °C. Thus, heat generation by the MDC sponges was controlled by switching the AMF on and off.

Although the MDC sponges released no DOX before the first AMF application, they started releasing DOX immediately after the AMF was switched on (Figure 6B). When AMF application was stopped, DOX release from the MDC sponges became slow. When AMF was applied again, a fast DOX release from the MDC sponges was again observed. A similar DOX release response was observed as the AMF was subsequently turned on and off. The above results demonstrated that both the heat generation ability of the MDC sponges and DOX release from them were remotely and actively controlled by switching the AMF on and off.

### 3.6. The Tumor Cell Killing Ability of the MDC Sponges

**Figure 7:**
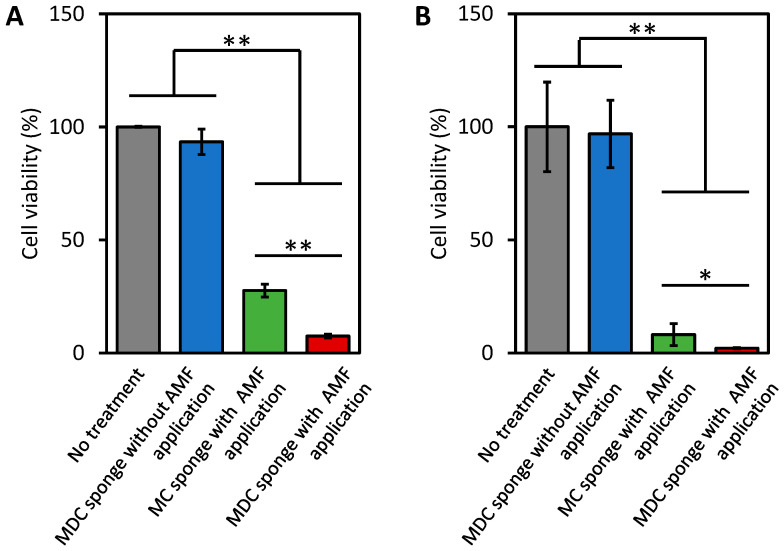
Viability of untreated HeLa cells incubated without sponges and AMF application, with the MDC sponge and no AMF application, with MC sponge (without DOX) and 15-min AMF application, and with MDC sponge and 15-min AMF application at 3 (**A**) and 5 (**B**) days, respectively (* *p* < 0.05 and ** *p* < 0.01).

The viability of the tumor cells (HeLa cells) that were incubated with the MDC sponge in the absence of AMF exposure for 3 and 5 days was approximately 100% (Figure 7A,B), suggesting that there was no DOX release from the MDC sponge. The viabilities of cells that were subjected to a 15-min AMF application in the presence of the MC sponge and subsequently incubated with the MC sponge in the absence of AMF at days 3 and 5 were 27.6% ± 2.8% and 8.1% ± 4.9%, respectively (Figure 7A,B). Thus, thermal treatment was effective in killing tumor cells. To evaluate the effects of combined thermal treatment and chemotherapy, the cells were subjected to a 15-min AMF application in the presence of the MDC sponge. Cell viabilities at days 3 and 5 were 7.5% ± 0.8% and 2.1% ± 0.3%, respectively (Figure 7A,B). These results demonstrated that the combination of thermal treatment and DOX action led to higher tumor cell killing effects than thermal treatment alone. Furthermore, gradual DOX release from the MDC sponges after switching the AMF off continued to promote tumor cell killing and cell growth inhibition. Thus, with a simple surgical procedure, the MDC sponges may be utilized to achieve on-demand treatment and subsequent remotely controlled administration of efficient treatments.

Currently, collagen sponges are used in clinical practice due to their handleability. For example, Teruplug^®^ is filled into tooth extraction wounds for hemostasis, the protection of the wound surface, and the promotion of tissue formation [50]. This fact suggests that MDC sponges, which have favorable handleability, are considered applicable in clinical practice.

## 4. Conclusions

This study successfully produced tearable MDC sponges. The degree of crosslinking of the MDC sponges was controlled by adjusting the crosslinking time. A 6-h crosslinking prevented undesirable DOX release from the MDC sponges in the absence of AMF exposure, but it allowed DOX release after AMF exposure. Heat generation and DOX release were controlled by switching the AMF on and off. Furthermore, AMF application in the presence of the MDC sponges had profound effects on destroying tumor cells, and the effect continued after termination of AMF exposure. Although the present study evaluated the handleability and cell killing abilities of MDC sponges through in vitro experiments, in vivo experiments are necessary for the evaluation of practical usefulness. Therefore, in future studies, we will evaluate the usefulness of MDC sponges through in vivo experiments.

## Supplementary Materials

The following are available online at https://www.mdpi.com/1996-1944/13/16/3637/s1, Figure S1: XRD patterns of MNPs, Figure S2: Relationship between crystallite size and Fe(acac)_3_ concentration, Figure S3: Magnetization curves of MNPs at room temperature, Figure S4: Photograph and thermal images of MNPs-8 dispersed in distilled water before and during AMF application, Figure S5: FTIR spectra of MDC sponges before and after crosslinking for 1.5, 6, and 24 h. Table S1: Synthesis conditions of MNPs.

## Abbreviations

AMF	alternating magnetic field
MNPs	magnetite nanoparticles
DOX	doxorubicin hydrochloride
FTIR	Fourier-transform infrared
MDC	MNPs and DOX-loaded collagen sponge
SEM	scanning electron microscope
TEM	transmission electron microscope

## References

[1] Weller M., van den Bent M., Tonn J.C., Stupp R., Preusser M., Cohen-Jonathan-Moyal E., Henriksson R., Le Rhun E., Balana C., Chinot O. (2017). European Association for Neuro-Oncology (EANO) guideline on the diagnosis and treatment of adult astrocytic and oligodendroglial gliomas. Lancet Oncol..

[2] Harbeck N., Gnant M. (2017). Breast cancer. Lancet.

[3] Tetsworth K., Block S., Glatt V. (2017). Putting 3D modelling and 3D printing into practice: Virtual surgery and preoperative planning to reconstruct complex post-traumatic skeletal deformities and defects. J. Soc. Int. Chir. Orthop. Traumatol..

[4] Brennan T., Tham T.M., Costantino P. (2017). The Temporalis Muscle Flap for Palate Reconstruction: Case Series and Review of the Literature. Int. Arch. Otorhinolaryngology.

[5] Chan D.S., Fnais N., Ibrahim I., Daniel S., Manoukian J. (2019). Exploring polycaprolactone in tracheal surgery: A scoping review of in-vivo studies. Int. J. Pediatr. Otorhinolaryngol..

[6] Palamà I.E., Arcadio V., D’Amone S., Biasiucci M., Gigli G., Cortese B. (2017). Therapeutic PCL scafold for reparation of resected osteosarcoma defect. Sci. Rep..

[7] Sarkar N., Bose S. (2019). Liposome-Encapsulated Curcumin-Loaded 3D Printed Scaffold for Bone Tissue Engineering. ACS Appl. Mater. Interfaces.

[8] Chew S.A., Danti S. (2017). Biomaterial-Based Implantable Devices for Cancer Therapy. Adv. Healthc. Mater..

[9] Huang W.W., Ling S.J., Li C.M., Omenetto F.G., Kaplan D.L. (2018). Silkworm silk-based materials and devices generated using bio-nanotechnology. Chem. Soc. Rev..

[10] Farid-ul-Haq M., Haseeb M.T., Hussain M.A., Ashraf M.U., Naeem-ul-Hassan M., Hussain S.Z., Hussain I. (2020). A smart drug delivery system based on Artemisia vulgaris hydrogel: Design, on-off switching, and real-time swelling, transit detection, and mechanistic studies. J. Drug Deliv. Sci. Technol..

[11] Yazdi M.K., Zarrintaj P., Hosseiniamoli H., Mashhadzadeh A.H., Saeb M.R., Ramsey J.D., Ganjali M.R., Mozafari M. (2020). Zeolites for theranostic applications. J. Mater. Chem. B.

[12] Lu H., Zhang N., Ma M.M. (2019). Electroconductive hydrogels for biomedical applications. Wiley Interdiscip. Rev.-Nanomed. Nanobiotechnology.

[13] Robert M.C., Frenette M., Zhou C., Yan Y., Chodosh J., Jakobiec F.A., Stagner A.M., Vavvas D., Dohlman C.H., Paschalis E.I. (2016). A Drug Delivery System for Administration of Anti-TNF-alpha Antibody. Transl. Vis. Sci. Technol..

[14] Pawar V., Bulbake U., Khan W., Srivastava R. (2019). Chitosan sponges as a sustained release carrier system for the prophylaxis of orthopedic implant-associated infections. Int. J. Biol. Macromol..

[15] Pritchard E.M., Valentin T., Panilaitis B., Omenetto F., Kaplan D.L. (2013). Antibiotic-Releasing Silk Biomaterials for Infection Prevention and Treatment. Adv. Funct. Mater..

[16] Zhang Z.Y., Kuang G.Z., Zong S., Liu S., Xiao H.H., Chen X.S., Zhou D.F., Huang Y.B. (2018). Sandwich-Like Fibers/Sponge Composite Combining Chemotherapy and Hemostasis for Efficient Postoperative Prevention of Tumor Recurrence and Metastasis. Adv. Mater..

[17] Ibrahim H.K., Fahmy R.H. (2016). Localized rosuvastatin via implantable bioerodible sponge and its potential role in augmenting bone healing and regeneration. Drug. Deliv..

[18] Cen D., Wan Z., Fu Y.K., Pan H.Q., Xu J.J., Wang Y.F., Wu Y.J., Li X., Cai X.J. (2020). Implantable fibrous ‘patch’ enabling preclinical chemo-photothermal tumor therapy. Colloid Surf. B-Biointerfaces.

[19] Perez-Martinez C.J., Chavez S.D.M., del Castillo-Castro T., Ceniceros T.E.L., Castillo-Ortega M.M., Rodriguez-Felix D.E., Ruiz J.C.G. (2016). Electroconductive nanocomposite hydrogel for pulsatile drug release. React. Funct. Polym..

[20] Shah S.A.A., Firlak M., Berrow S.R., Halcovitch N.R., Baldock S.J., Yousafzai B.M., Hathout R.M., Hardy J.G. (2018). Electrochemically Enhanced Drug Delivery Using Polypyrrole Films. Materials.

[21] Leary M., Heerboth S., Lapinska K., Sarkar S. (2018). Sensitization of Drug Resistant Cancer Cells: A Matter of Combination Therapy. Cancers.

[22] Sanai N., Berger M.S. (2018). Surgical oncology for gliomas: The state of the art. Nat. Rev. Clin. Oncol..

[23] Nault J.C., Sutter O., Nahon P., Ganne-Carrie N., Seror O. (2018). Percutaneous treatment of hepatocellular carcinoma: State of the art and innovations. J. Hepatol..

[24] Chen J.J., Wang Y.T., Ma B.Y., Guan L., Tian Z.F., Lin K.L., Zhu Y.F. (2020). Biodegradable hollow mesoporous organosilica-based nanosystems with dual stimuli-responsive drug delivery for efficient tumor inhibition by synergistic chemo- and photothermal therapy. Appl. Mater. Today.

[25] Fearon K., Strasser F., Anker S.D., Bosaeus I., Bruera E., Fainsinger R.L., Jatoi A., Loprinzi C., MacDonald N., Mantovani G. (2011). Definition and classification of cancer cachexia: An international consensus. Lancet Oncol..

[26] Lin Y., Zhang K., Zhang R., She Z., Tan R., Fan Y., Li X. (2020). Magnetic nanoparticles applied in targeted therapy and magnetic resonance imaging: Crucial preparation parameters, indispensable pre-treatments, updated research advancements and future perspectives. J. Mater. Chem. B.

[27] Shasha C., Krishnan K.M. (2020). Nonequilibrium Dynamics of Magnetic Nanoparticles with Applications in Biomedicine. Adv. Mater..

[28] Wang X., Law J., Luo M., Gong Z., Yu J., Tang W., Zhang Z., Mei X., Huang Z., You L. (2020). Magnetic Measurement and Stimulation of Cellular and Intracellular Structures. ACS Nano.

[29] Israel L.L., Galstyan A., Holler E., Ljubimova J.Y. (2020). Magnetic iron oxide nanoparticles for imaging, targeting and treatment of primary and metastatic tumors of the brain. J. Control. Release.

[30] Xiao Y., Du J. (2020). Superparamagnetic nanoparticles for biomedical applications. J. Mater. Chem. B.

[31] Hayashi K., Sato Y., Sakamoto W., Yogo T. (2017). Theranostic Nanoparticles for MRI-Guided Thermochemotherapy: “Tight” Clustering of Magnetic Nanoparticles Boosts Relaxivity and Heat-Generation Power. ACS Biomater. Sci. Eng..

[32] Hayashi K., Sakamoto W., Yogo T. (2016). Smart Ferrofluid with Quick Gel Transformation in Tumors for MRI-Guided Local Magnetic Thermochemotherapy. Adv. Funct. Mater..

[33] Pucci C., De Pasquale D., Marino A., Martinelli C., Lauciello S., Ciofani G. (2020). Hybrid Magnetic Nanovectors Promote Selective Glioblastoma Cell Death through a Combined Effect of Lysosomal Membrane Permeabilization and Chemotherapy. ACS Appl. Mater. Interfaces.

[34] Chen W., Cheng C.A., Zink J.I. (2019). Spatial, temporal, and dose control of drug delivery using noninvasive magnetic stimulation. ACS Nano.

[35] Hayashi K., Sakamoto W., Yogo T. (2009). Magnetic and rheological properties of monodisperse Fe_3_O_4_ nanoparticle/organic hybrid. J. Magn. Magn. Mater..

[36] Hayashi K., Sakamoto W., Yogo T. (2013). One-pot synthesis of magnetic nanoparticles assembled on polysiloxane rod and their response to magnetic field. Colloid Polym. Sci..

[37] Latham A.H., Williams M.E. (2008). Controlling Transport and Chemical Functionality of Magnetic Nanoparticles. Acc. Chem. Res..

[38] Jeong U., Teng X.W., Wang Y., Yang H., Xia Y.N. (2007). Superparamagnetic colloids: Controlled synthesis and niche applications. Adv. Mater..

[39] Hergt R., Andrä W., d’Ambly C.G., Hilger I., Kaiser W.A., Richter U., Schmidt H.-G. (1998). Physical Limits of Hyperthermia Using Magnetite Fine Particles. IEEE Trans. Magn..

[40] Timko M., Dzarova A., Kovac J., Skumiel A., Józefczak A., Hornowski T., Gojżewski H., Zavisova V., Koneracka M., Sprincova A. (2009). Magnetic properties and heating effect in bacterial magnetic nanoparticles. J. Magn. Magn. Mater..

[41] Drake P., Cho H.J., Shih P.S., Kao C.H., Lee K.F., Kuo C.H., Lin X.Z., Lin Y.J. (2007). Gd-doped iron-oxide nanoparticles for tumour therapy via magnetic field hyperthermia. J. Mater. Chem..

[42] Hiergeist R., Andrä W., Buske N., Hergt R., Hilger I., Richter U., Kaiser W. (1999). Magnetic properties and heating effect in bacterial magnetic nanoparticles. J. Magn. Magn. Mater..

[43] Hayashi K., Moriya M., Sakamoto W., Yogo T. (2009). Chemoselective Synthesis of Folic Acid-Functionalized Magnetite Nanoparticles via Click Chemistry for Magnetic Hyperthermia. Chem. Mater..

[44] Ruszczak Z., Friess W. (2003). Collagen as a carrier for on-site delivery of antibacterial drugs. Adv. Drug Deliv. Rev..

[45] Angele P., Abke J., Kujat R., Faltermeier H., Schumann D., Nerlich M., Kinner B., Englert C., Ruszczak Z., Mehrl R. (2004). Influence of different collagen species on physico-chemical properties of crosslinked collagen matrices. Biomaterials.

[46] Fernandes L.L., Resende C.X., Tavares D.S., Soares G.A., Castro L.O., Granjeiro J.M. (2011). Cytocompatibility of Chitosan and Collagen-Chitosan Scaffolds for Tissue Engineering. Polimeros.

[47] Kanwal U., Bukhari N.I., Rana N.F., Rehman M., Hussain K., Abbas N., Mehmood A., Raza A. (2019). Doxorubicin-loaded quaternary ammonium palmitoyl glycol chitosan polymeric nanoformulation: Uptake by cells and organs. Int. J. Nanomed..

[48] Madaghiele M., Calo E., Salvatore L., Bonfrate V., Pedone D., Frigione M., Sannino A. (2016). Assessment of collagen crosslinking and denaturation for the design of regenerative scaffolds. J. Biomed. Mater. Res. Part A.

[49] Hayashi K., Nakamura M., Sakamoto W., Yogo T., Miki H., Ozaki S., Abe M., Matsumoto T., Ishimura K. (2013). Superparamagnetic Nanoparticle Clusters for Cancer Theranostics Combining Magnetic Resonance Imaging and Hyperthermia Treatment. Theranostics.

[50] Hur J.-W., Yoon S.-J., Ryu S.-Y. (2012). Comparison of the bone healing capacity of autogenous bone, demineralized freeze dried bone allograft, and collagen sponge in repairing rabbit cranial defects. J. Korean Assoc. Oral Maxillofac. Surg..

