# Supplementary material for: Tearable and Fillable Composite Sponges Capable of Heat Generation and Drug Release in Response to Alternating Magnetic Field
Source: Materials (Basel). 2020 Aug 17;13(16):3637. doi: 10.3390/ma13163637 (PMC7475967; doi:10.3390/ma13163637)
Supplement: Supplementary file 1 [file materials-13-03637-s001.pdf]

materials

MDPI

Suplementary Materials

Table S1. Synthesis conditions of MNPs.

Sample	$Fe(acac)_3$ concentration (mM)	Additive Amount of Hydrazine Monohydrate (equiv.)	Additive Amount of Distilled Water (equiv.)
MNPs-1	0.5	6	72
MNPs-2	0.7	6	72
MNPs-3	0.9	4	72
MNPs-4	0.9	6	36
MNPs-5	0.9	6	72
MNPs-6	0.9	6	108
MNPs-7	0.9	8	72
MNPs-8	0.9	8	144
MNPs-9	1.1	6	72

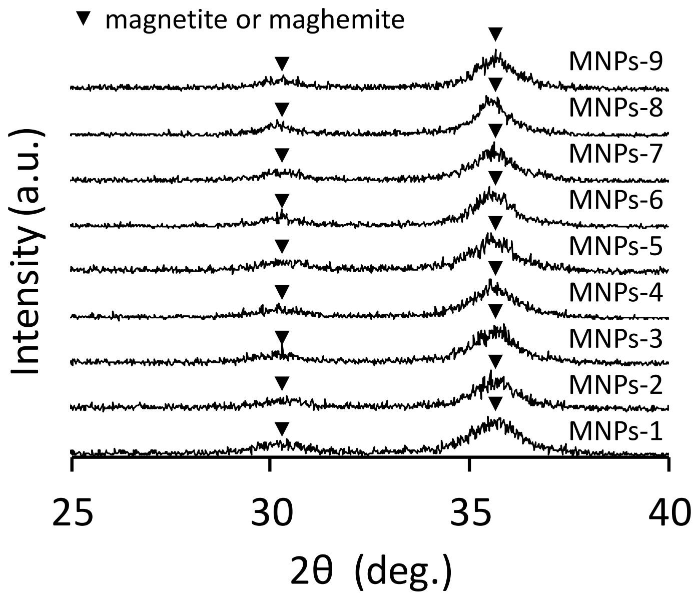

Figure S1. XRD patterns of MNPs.

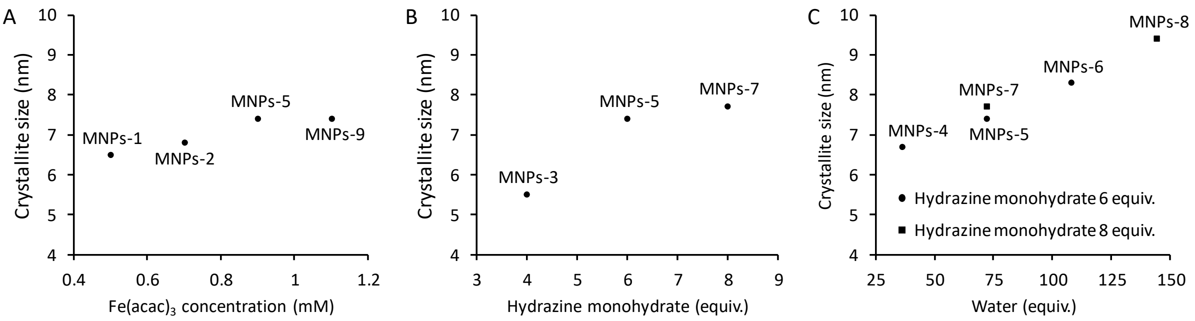

Figure S2. Relationship between crystallite size and Fe(acac)3 concentration (**A**), the molar equivalent of hydrazine monohydrate to Fe(acac)3 (**B**), or the molar equivalent of water to Fe(acac)3 (**C**).

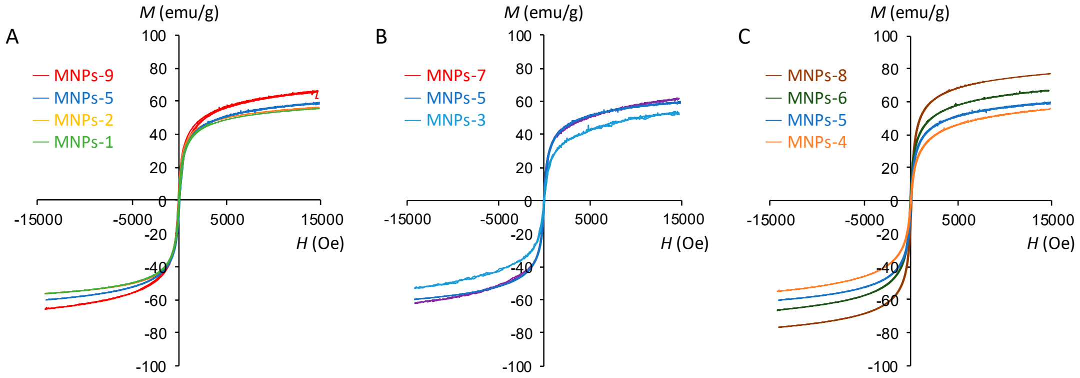

Figure S3. Magnetization curves of MNPs at room temperature. The influences of Fe(acac)3 concentration (**A**), additive amounts of hydrazine monohydrate (**B**), and water (**C**) on magnetic properties.

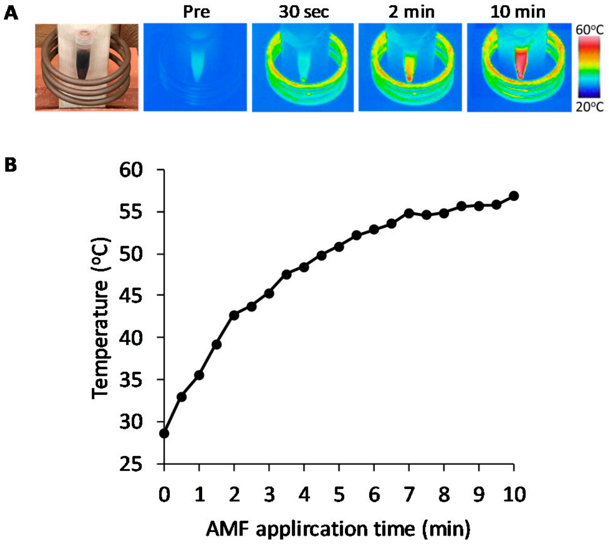

Figure S4. (**A**) Photograph and thermal images of MNPs-8 dispersed in distilled water before and during AMF application. (**B**) Temperature change of MNPs-8-dispersed water following AMF application.

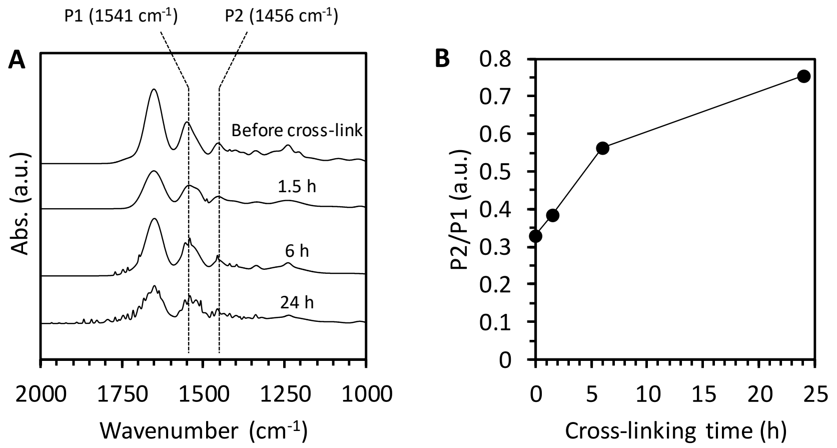

Figure S5. (**A**) FTIR spectra of MDC sponges before and after crosslinking for 1.5, 6, and 24 h. (**B**) Effects of crosslinking time on the area ratio of P2 to P1, which indicates crosslinking density.
